# Is there a difference in rhythm outcome between patients undergoing first line versus second line paroxysmal atrial fibrillation ablation?

**DOI:** 10.1371/journal.pone.0208994

**Published:** 2018-12-07

**Authors:** Martin Manninger, Jakob Ebner, David Zweiker, Raphael Sieghartsleitner, Bernadette Mastnak, Egbert Bisping, Peter Lercher, Rita Riedlbauer, Brigitte Rotman, Helmut Brussee, Daniel Scherr

**Affiliations:** 1 Division of Cardiology, Department of Internal Medicine, Medical University of Graz, Graz, Austria; 2 Department of Cardiology, Cardiovascular Research Institute Maastricht (CARIM), Maastricht University Medical Center, Limburg, The Netherlands; University of Minnesota, UNITED STATES

## Abstract

**Background:**

Catheter ablation of atrial fibrillation (AF) is an established second line therapy for patients with symptomatic paroxysmal AF (PAF) and may be considered as a first line therapy in selected patients who are highly symptomatic, considering patient choice, benefit, and risk, according to recent guidelines. Our study investigated whether a first line vs. second line ablation approach may result in improved sinus rhythm maintenance after ablation.

**Methods:**

A total of 153 patients undergoing pulmonary vein isolation for PAF were included in the study (age 55±12 years, 29% female). Seventy-nine patients underwent first line AF ablation and 74 patients underwent second line AF ablation after failed antiarrhythmic drug therapy. There was no significant difference in baseline characteristics such as age, history of AF, left atrial size or LVEF between groups. Success was defined as atrial tachyarrhythmia free survival during a 12-month follow-up by means of serial ECG Holter monitoring.

**Results:**

There was no significant difference in cumulative arrhythmia-free survival between those patients who received AF ablation as a first or second line therapy. Single procedure success was 78% in the first line group vs. 81% in the second line group; multiple procedure success was 90 vs. 91%, (n.s.). Complication rate was 1.3% vs. 1.4% (n.s.).

**Conclusion:**

Success of AF ablation did not differ between patients who receive ablation as first vs. second line therapy. Based on these data, a trial of AAD therapy before AF ablation may be justified in most patients with symptomatic PAF eligible for rhythm control.

## Introduction

Catheter ablation of paroxysmal atrial fibrillation (PAF) is an established treatment option for symptomatic AF patients. Recent ESC guidelines and HRS/EHRA/ECAS/APHRS/SOLAECE consensus statement recommend catheter ablation as a rhythm control therapy to improve symptoms in patients with symptomatic AF recurrences despite antiarrhythmic drug (AAD) therapy. [1, 2] Considering patient choice as well as benefit and risk of an invasive treatment, catheter ablation can be considered as a first-line therapy before starting AAD therapy.

While several randomized controlled trials have compared the efficacy of first [3–6] or second [7–9] line catheter ablation to AAD therapy, there is paucity of data comparing outcomes after catheter ablation of PAF as an initial therapy versus after failed AAD therapy.

Regarding the well-known principle that AF begets AF [10] and the fact that catheter ablation has been shown to be a more effective rhythm control therapy as compared to AAD therapy [11], early ablation could be beneficial for arrhythmia free survival. In retrospective analyses, ablation within one year after diagnosis of AF has been shown to increase arrhythmia-free survival. [12] On the other hand, waiting periods for catheter ablation are long in daily clinical practice and could be bridged with antiarrhythmic drugs to allow temporary rhythm control and therefore prevent disease progression. [13, 14]

Therefore, we investigated whether a first line vs. second line ablation approach may result in improved sinus rhythm maintenance in paroxysmal AF patients.

## Materials and methods

### Study population

A total of 153 consecutive symptomatic patients undergoing pulmonary vein isolation for PAF from 2012 to 2016 were retrospectively included in the study. Initial decision for initiation of AAD therapy was taken by the admitting physician which determined inclusion in either group. Failed AAD therapy was defined as the occurrence of symptomatic episodes of AF during treatment with a maximum of two different class I or III AADs, after discontinuation of AAD therapy due to side effects after a minimum AAD treatment duration of one month or after switching of AADs due to symptomatic recurrences after a trial of AAD for a minimal period of 1 month. This study was approved by the ethics committee of the Medical University of Graz and all patients gave written informed consent.

### Electrophysiological study and ablation procedure

Patients received oral anticoagulation with vitamin K antagonists (VKA) or non-VKA oral anticoagulants for at least four weeks prior to the procedure depending on their CHA_2_DS_2_-VASc scores. Patients underwent transoesophageal echocardiography within 48h before the procedure to rule-out atrial thrombus. Immediately before transseptal puncture, intravenous heparin (100IU/kg) was administered, and was repeated during the procedure depending on regular measurements of activated clotting times (ACT), with a target ACT of 300-400sec. The ablation procedure was guided by three-dimensional electroanatomic mapping (CARTO 3, Biosense Webster). Catheter ablation of the pulmonary veins (PVs) was performed using a 3.5 mm irrigated tip catheter (ThermoCool SmartTouch, Biosense Webster) with a standard power setting of 30W for the anterior portions of the atrium and 25W for the remaining left atrium. A 10-polar lasso catheter (CARTO Lasso, Biosense Webster) was used for fast anatomical mapping and to control entrance and exit block within all four PVs. Wide antral circumferential contact force guided PV isolation (PVI) was performed with the endpoint of abolition or dissociation of electric activity of all PVs. Successful PVI was checked after a 20-minute waiting period including sinus rhythm control and entrance-block pacing. None of the patients underwent more extensive ablations (such as linear ablation, ablation of complex fractionated electrograms, rotor ablation). In case of documented typical right atrial flutter, additional cavotricuspid isthmus ablation was performed during CS pacing until bidirectional block was achieved.

Anticoagulation was restarted the day of the procedure for at least three months after the ablation procedure and was continued thereafter at the physician’s discretion.

### Follow-up

Patients were followed up at our institution or at their referring physicians 3, 6, and 12 months after the procedure including 24h Holter monitoring. Between regular visits, all patients were encouraged to seek ECG or Holter monitoring for any symptoms suggestive of AF.

In second line ablation patients, AADs were continued for at least 3 months after the ablation procedure and were stopped thereafter at the physician’s discretion. In first line ablation patients AAD treatment was commenced after the ablation for at least 3 months after the ablation procedure and were stopped thereafter at the physician’s discretion. Repeat ablation was offered to patients with arrhythmia recurrence after the initial 3-month follow-up period. The primary study endpoint was freedom from any asymptomatic or symptomatic atrial tachyarrhythmia lasting >30 s after the initial ablation procedure.

In case of AF recurrence, patients underwent re-do ablations, which consisted of a re-do wide antral pulmonary vein isolation with the endpoint of abolition or dissociation of electric activity of all PVs, and/or of an ablation of atrial tachycardia or atrial flutter if present. Follow-up duration was reset after a re-do ablation.

### Data processing and statistical analyses

Continuous variables are presented as mean±SD or median (interquartile range). Categorical variables are presented as percentages (%) and counts. Two-group comparisons of continuous variables were performed by Student’s t tests if normally distributed or with Wilcoxon rank-sum tests if the normality assumption was violated according to Shapiro-Wilk tests or visual inspection of normal probability plots. Categorical variables were compared by chi-square tests or Fisher’s exact test, respectively. Time to first arrhythmia recurrence was calculated and plotted using the Kaplan–Meier product-limit method with comparisons performed by log-rank statistics. Two-tailed P values <0.05 were considered to indicate statistical significance.

Baseline characteristics, including age, sex, comorbidities, and pharmacological therapy, were complete in all patients. Statistical analyses were performed using SPSS 23.0 (IBM, Armonk, NY).

## Results

Seventy-nine patients (52%) underwent first line AF ablation, while 74 patients (48%) underwent second line AF ablation after 1.1±0.3 trials of failed AAD therapy. Baseline characteristics and inter-group comparisons are displayed in Table 1. Out of patients who underwent ablation after failed AAD treatment, 57% previously received class I (propafenone—minimal dosage of 150mg b.d.: n = 36, flecainide—minimal dosage of 100mg b.d.: n = 6) and 51% class III (amiodarone—minimal daily dosage of 200mg: n = 30, dronedarone—minimal daily dosage of 400mg b.d.: n = 8). AAD therapy prior to ablation is displayed in Table 2.

**Table 1:** Baseline characteristics of patients who underwent ablation as a first or second line therapy with inter-group comparison.

		first line (n = 79)	second line (n = 74)	p-value
**age (y)**		56 (48, 68)	57 (41, 64)	0.22
**female sex (%)**		30%	27%	0.72
**BMI**		26.2±3.4	28.2±4.5	0.003
**medical history**	CAD	10 (13%)	8 (11%)	0.81
	HT	39 (49%)	50 (68%)	0.033
	prior stroke / TIA	3 (4%)	2 (3%)	1.00
	diabetes	3 (4%)	6 (8%)	0.32
	CHADS_2_-Score	1 (0, 1)	1 (0, 1)	0.052
	CHA_2_DS_2_-VASc-Score	1 (0, 2)	2 (1, 2)	0.08
**echocardiography**	LVEF (%)	62 (60, 63)	65 (58, 66)	0.86
	LVEDD (mm)	50 (49, 52)	48 (42, 53)	0.26
	LA diameter (mm)	51±5	52±7	0.79
**AF history (months)**		36 (23, 84)	36 (31, 168)	0.43
**time from presentation to ablation (months)**		2.35±1.5	2.15±1.3	0.38
**lab values**	nt-pro-BNP (ng/L)	139 (54, 365)	216 (63, 176)	0.30
	creatinine (mg/dL)	0.96 (0.78, 1.0)	0.96 (0.84, 1.15)	0.81
	GFR (mL/min)	80.2±19	78.8±19	0.65

BMI = body mass index, CAD = coronary artery disease, HT = arterial hypertension, TIA = transitory ischemic attack, LVEF = left ventricular ejection fraction, LVEDD = left ventricular end diastolic diameter, AF = atrial fibrillation, nt-pro-BNP = b-type brain natriuretic peptide, GFR = glomerular filtration rate calculated with CKD-EPI-formula.

**Table 2:** Antiarrhythmic drug therapy prior to AF ablation.

	first line (n = 79)	second line (n = 74)	p-value
**Beta blockers**	55 (70%)	53 (72%)	0.31
**Calcium channel blockers**	0 (0%)	1 (1%)	1.00
**Class I antiarrhythmic drugs**	0 (0%)	42 (57%)	n/a
**Class III antiarrhythmic drugs**	0 (0%)	38 (52%)	n/a

There was no significant difference between groups in baseline characteristics such as age, gender, CHADS_2_-score, CHA_2_DS_2_-VASc-Score, history of AF, waiting time to AF ablation, left atrial (LA) size, left ventricular end diastolic diameter (LVEDD) or left ventricular ejection fraction (EF) between groups (Table 1). Patients undergoing ablation as a second line treatment had a higher BMI (28.2±4.5 vs. 26.2±3.4, p = 0.003) and a higher prevalence of arterial hypertension (68% vs. 49%, p = 0.033). Laboratory values upon admission to the ablation treatment including nt-pro-BNP, creatinine and GFR calculated with the CKD-EPI formula were comparable between both groups.

Twenty percent of patients undergoing first line ablation and 32% of second line patients had prior medical or electrical cardioversions within 7 days of AF onset (p = n.s.).

### Index procedure

Successful PVI checked by entrance- and exit-block pacing after a 20-minute waiting period was achieved in 100% of all patients. In 15% of first line patients and 20% of second line patients, additional cavo-tricuspid isthmus ablation was performed in the same procedure due to documented common-type right atrial flutter (p = n.s.) which was successful in all patients. Mean fluoroscopy time was 35±17 min in first line patients and 37±15 min in second line patients (p = n.s.). Median radiofrequency time was 38 (32, 40) min in first line patients and 38 (31, 42) min in second line patients (p = n.s.). Ablation procedures lasted 147 (141, 196) min in first line patients and 153 (136, 157) min in second line patients (p = n.s.). A summary of procedural outcomes and complications is displayed in Table 3.

**Table 3:** Outcomes after ablation.

	first line (n = 79)	second line (n = 74)	p-value
**Single procedure success**	62 (78%)	60 (81%)	0.82
**Multiple procedure success**	71 (90%)	67 (91%)	0.35
**Re-do-ablations**	7 (9%)	11 (15%)	0.32
**Complications**	1 (1.3%)	1 (1.4%)	1.00
**Patients on AAD at last visit**	23 (29%)	21 (28%)	0.53

There was no significant difference between single/multiple procedure success, re-do-ablations, complications and AAD therapy between groups.

### Complications

Intraprocedural complications occurred in 1 patient in each group (1.3 vs. 1.4%). One patient who underwent ablation as a first line therapy suffered from an inguinal aneurysm after ablation which required surgical treatment.

One patient in the second line group suffered from ischemic stroke three days after the ablation treatment although being on oral anticoagulation with rivaroxaban.

### Redo-ablations

After recurrence within the first 12 months of follow-up, 7 first line patients (9%) and 11 second line patients (15%) underwent a second ablation due to AF in 5 vs. 9 patients and atrial flutter in 2 vs. 2 patients (p = n.s.). In all redo procedures, PV reconnection was discovered and successfully ablated. In patients with pulmonary vein dependant atrial flutter (1 vs. 2), PVI successfully terminated atrial flutter, in one patient, mitral isthmus dependant atrial flutter was terminated with mitral isthmus ablation. Primary success in redo ablations, defined by complete PVI and/or termination of atrial flutter including bidirectional linear block, was achieved in 100% of patients.

### Outcome

The median follow-up time was 352 (198, 365) days in first line patients vs. 356 (135, 365) days in second line patients (n.s.).

**Fig 1:**
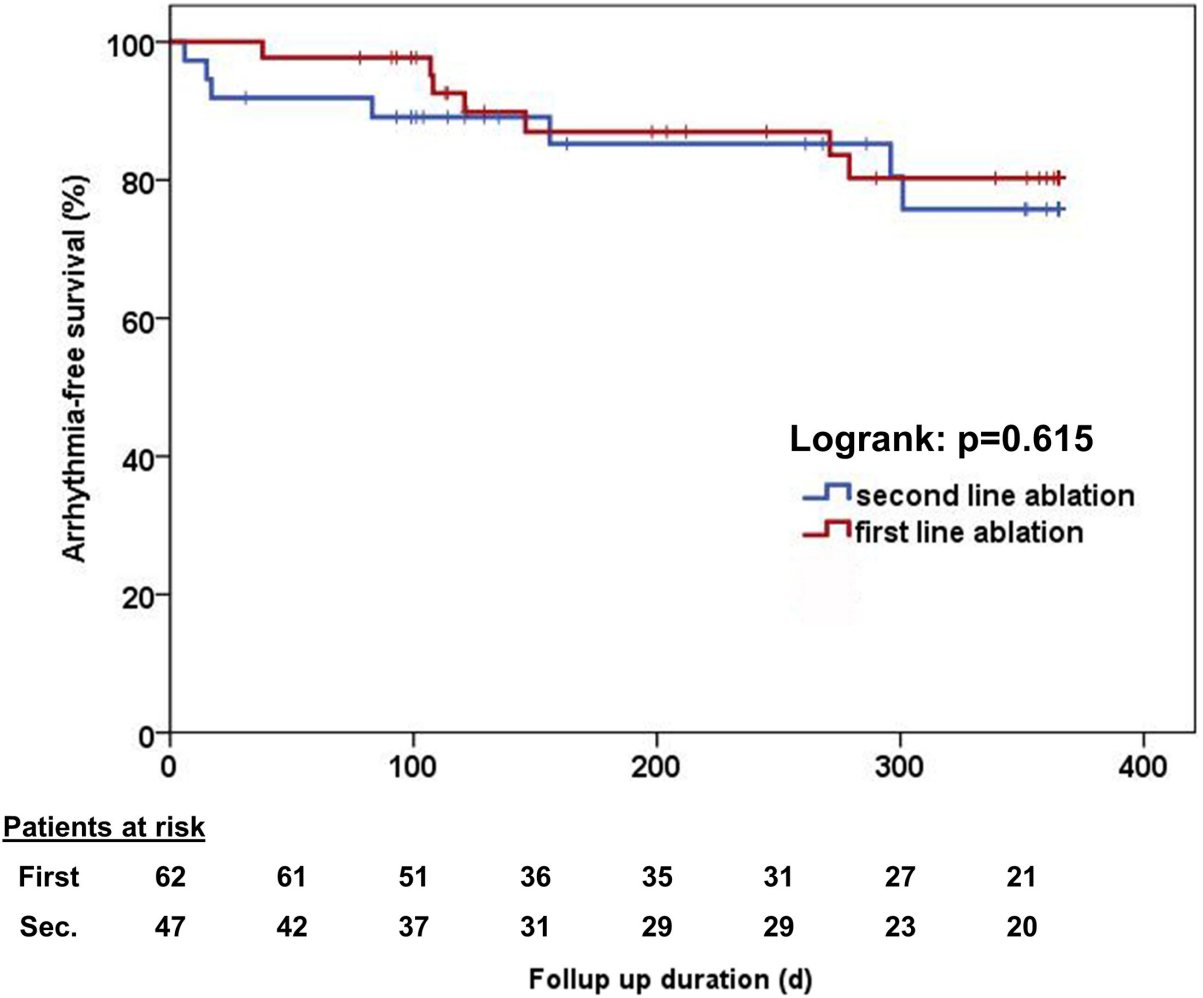
Single procedure success rate off antiarrhythmic drugs. There was no significant difference between patients who received ablation as a first or second line therapy (Log rank test: p = 0.615).

There was no significant difference in cumulative arrhythmia-free survival between those patients who received AF ablation as a first or second line therapy (single procedure success 78% in the first line group vs. 81% in the second line group; Log rank test p = 0.535). There was no difference in single procedure outcome off AADs between both groups (79% vs. 83%; Log rank test p = 0.615, Fig 1).

**Fig 2:**
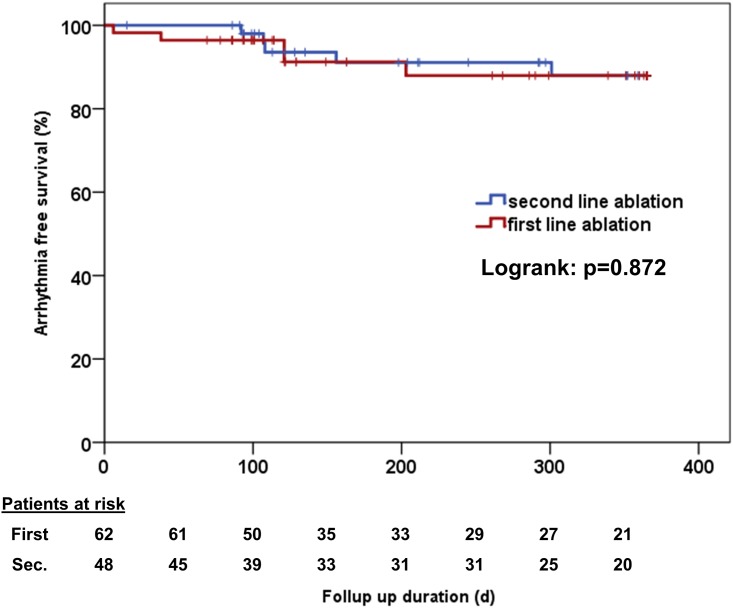
Multiple procedure success rate on and off antiarrhythmic drugs. There was no significant difference between patients who received ablation as a first or second line therapy (Log rank test: 0.872).

Multiple procedure success rate was 90 vs. 91% (Log rank test p = 0.674) and multiple procedure success rate off AADs was 90% vs. 91% (Log rank test p = 0.872, Fig 2). Median time to recurrence was 100 (26, 175) days in first line patients and 123 (56, 296) days in second line patients (p = 0.42). Twenty-nine percent of first line patients compared to 28% of second line patients were still on AAD therapy at the last visit (p = 0.53).

Seventeen first line and 14 second line patients had recurrences during follow-up. Thirteen vs. 12 patients experienced paroxysmal AF, 1 vs. 1 patients persistent AF and 3 vs. 1 patients atrial tachycardia (p = 0.69).

## Discussion

Our study demonstrates that in patients with PAF, a second-line ablation strategy after a trial of antiarrhythmic drug treatment is non-inferior to a direct first line ablation strategy over a 12-month follow-up period.

Current guidelines recommend catheter ablation of symptomatic paroxysmal AF as a second line therapy in patients after failed AAD treatment. [1, 2] This recommendation is based on one randomized controlled trial as well as a meta-analysis comparing outcome of second line AF ablation and AAD therapy, both of which showed higher arrhythmia-free survival after AF ablation. [7, 8]

MANTRA-PAF, a multicentre randomized controlled trial, had set the scene for AF ablation as a first line therapy, showing that reduction of AF burden was comparable for AF ablation and antiarrhythmic drug therapy. [3] Five year follow-up in this study cohort revealed a reduction in AF burden and symptomatic recurrences as well as an improved quality of life. [15] These findings are reflected in current ESC and HRS/EHRA/ECAS/APHRS/SOLAECE guidelines, stating that in selected patients, taking into account patient choice, risk and benefit, catheter ablation can be considered as an alternative to initiating AAD therapy. [1, 2]

Both recommendations on second and first line ablation are based on studies comparing catheter ablation to AAD therapy. To date, there has been no study directly comparing outcome in patients undergoing first or second line ablation. We could show that outcome after ablation on and off AAD therapy was comparable, regardless whether patients were previously treated with an AAD.

These findings suggest that pre-treatment with AAD seems not to increase success of catheter ablation. This is in line with the finding from previous studies on antiarrhythmic drug therapy during the blanking period. Although short term AAD therapy is used to avoid AF recurrences after ablation, randomized controlled trials on improving long-term arrhythmia-free survival after drug therapy during the blanking period have been disappointing. [16, 17]

Although this study represents all-comers for ablation, both groups are comparable regarding patient’s characteristics. The only comorbidity that was more prevalent in the second line group was arterial hypertension. Nevertheless, the fact that echo parameters such as EF, LA size and LVEDD as well as nt-pro-BNP were comparable, indicates, that arterial hypertension was well-treated and not associated with an increase in remodelled substrate. Generally, as shown in other studies including patients with PAF, subjects had a small number of comorbidities. [18–20] Most patients in this study had CHADS_2_ and CHA_2_DS_2_-VASc-Scores of 1, only around one tenth had coronary artery disease and even less suffered from diabetes or had experienced a stroke or TIA.

The fact that outcome after ablation was not improved in second line patients raises the question, whether the waiting period to ablation should be bridged with AADs. AAD therapy increases sinus rhythm maintenance two-fold as compared to no therapy but might be pro-arrhythmic and frequently cause extra-cardiac side effects. [21–23] This fact must be considered in the patient population represented in this study that is in an early stage of AF with low AF burden, is relatively young and has few comorbidities.

Single as well as multiple procedure success rates in this patient cohort are comparable to prior studies. Since Ouyang and colleagues reported a one year success rate of 62% in 2010, multiple other groups have reported increasing single procedure success rates that are attributed to increasing experience and novel technologies including force measurements. [24] Introduction of contact force measurements increased one year arrhythmia-free survival to up to around 85% which is comparable to the 78 and 81% success rate reported in this trial. [18–20]

Results from this trial point into the same direction that remodelling in this patient cohort in PAF might not be so advanced that rhythm control with AAD therapy favours outcome. Keeping patients in the status of paroxysmal AF with infrequent episodes might be the cornerstone for arrhythmia-free survival. In this study it seems not to make a difference for one-year arrhythmia-free survival whether patients underwent a trial of AAD therapy or were ablated before an AAD trial, as long as time to ablation is not prolonged by an AAD trial, which has shown to be associated with poorer outcomes after ablation in prior studies. [25] This is also supported by the fact that both groups had comparable histories of AF and waiting time from presentation to AF ablation, so in our population, an AAD trial did not prolong time to ablation.

## Limitations

The current study describes results from a single center and included only a limited number of patients with few comorbidities. Therefore, outcomes may not be generalizable to all patients and ablation centers.

The study design relies on accurate recordkeeping and may include bias, therefore these findings need to be confirmed in a larger randomized controlled trial with a longer follow-up duration. Since decision upon initial AAD treatment was performed by the admitting physician, the study may also include selection bias. Furthermore, patients did not take their AADs prior ablation within a controlled trial and we relied on anamnesis for history of failed and/or discontinued AADs.

Despite extensive efforts to detect asymptomatic AF/AT recurrences, true recurrence rates may have been underestimated by a lack of detection of silent AF episodes.

## Conclusion

Success of AF ablation did not differ between patients who received AF ablation as first line therapy and those who received AF ablation as a second line therapy. Based on these data, a trial of AAD therapy before AF ablation may be justified in most patients with symptomatic PAF eligible for rhythm control.

## Supporting information

S1 Dataset Anonymized dataset. (XLSX)
